# Kinetic Monte Carlo Simulation Based Detailed Understanding of the Transfer Processes in Semi-Batch Iodine Transfer Emulsion Polymerizations of Vinylidene Fluoride

**DOI:** 10.3390/polym10091008

**Published:** 2018-09-10

**Authors:** Florian Brandl, Marco Drache, Sabine Beuermann

**Affiliations:** Institute of Technical Chemistry, Clausthal University of Technology, 38678 Clausthal-Zellerfeld, Germany; Florian.Brandl@tu-clausthal.de (F.B.); marco.drache@tu-clausthal.de (M.D.)

**Keywords:** vinylidene fluoride (VDF), semi-batch emulsion polymerization, iodine transfer polymerizations, kinetic Monte Carlo simulation

## Abstract

Semi-batch emulsion polymerizations of vinylidene fluoride (VDF) are reported. The molar mass control is achieved via iodine transfer polymerization (ITP) using IC_4_F_8_I as chain transfer agent. Polymerizations carried out at 75 °C and pressures ranging from 10 to 30 bar result in low dispersity polymers with respect to the molar mass distribution (MMD). At higher pressures a significant deviation from the ideal behavior expected for a reversible deactivation transfer polymerization occurs. As identified by kinetic Monte Carlo (kMC) simulations of the activation–deactivation equilibrium, during the initialization period of the chain transfer agent already significant propagation occurs due to the higher pressure, and thus, the higher monomer concentration available. Based on the kMC modeling results, semi-batch emulsion polymerizations were carried out as a two pressure process, which resulted in very good control of the MMD associated with a comparably high polymerization rate.

## 1. Introduction

Poly(vinylidene fluoride) (PVDF) is an important fluoropolymer due to its excellent chemical, thermal, and mechanical stability [1]. Moreover, it may crystallize in five different polymorphs [2,3]. The dipolar β phase is of particular interest, since it is the prerequisite for the ferro-, pyro- and piezoelectric properties of PVDF, which lead to a wide variety of advanced applications [4]. Optimizing the property control of the polymer architecture is of high importance. With respect to obtaining low dispersity molar mass distributions (MMD) and the possibility of synthesizing complex copolymer architectures e.g., block copolymers, the VDF monomer is more challenging than conventional (meth)acrylate monomers or styrene. Since the radical polymerization of VDF is associated with a primary propagating radical the methods typically employed in reversible deactivation transfer polymerization (RDTP), such as atom transfer radical polymerization (ATRP), nitroxide mediated polymerization (NMP), or reversible addition fragmentation transfer (RAFT) polymerization are not easily applied [5]. RAFT polymerizations constitute an exception as reported by the group of Ameduri, however, several challenges have to be overcome [6,7,8,9]. Iodine transfer polymerizations (ITP) belonging to the same group of degenerative transfer methods have proven to be very robust with respect to achieving good control of the MMD [10,11]. Still, information on the kinetics and mechanism of ITP for reactions leading to higher molar mass products are scarce. This is particularly true for ITP in emulsion at industrially relevant conditions.

A comprehensive review on the application of Monte Carlo methods in the field of polymer reaction engineering was provided by Brandão et al. [12]. An important advantage of kinetic Monte Carlo simulations of polymerization reactions is that detailed information on the microstructure of the polymers is accessible. KMC methods allow for the simulation of complex molar mass distributions under consideration of transfer [13,14,15] or crosslinking reactions [16] as well as chain length dependent termination [14]. Moreover, copolymerizations [17,18] and emulsion polymerizations may be modelled [19,20]. D’hooge et al. addressed the model-based design of the polymer microstructure for living polymerizations, reversible deactivation transfer polymerizations, and click chemistry [21]. KMC simulations were carried out for RDTP in general [22], ATRP [23], and NMP [24,25,26,27]. The method is particularly well suited for the treatment of the complex reaction mechanism associated with degenerative transfer polymerizations (RAFT) [28,29,30,31].

Recently, we reported on an exploratory study on semi-batch VDF emulsion polymerizations in conjunction with ITP to control MMDs [32]. While the feasibility of the experimental approach was shown, detailed information on the kinetics and mechanism of ITP under technically relevant conditions were not given. The aim of the current contribution is to derive chain transfer constants for the transformation of the initial chain transfer agent and for the main equilibrium involving the polymeric species. The experimental work is accompanied by kinetic Monte Carlo (kMC) simulations to obtain a better understanding of the underlying mechanism. In this contribution comparably facile kMC modeling is reported for ITP. The model applied allows for the description of the number average molar mass and the product distribution for a VDF semi-batch emulsion polymerization on the basis of two parameters.

## 2. Methods and Materials

### 2.1. Materials

Vinylidene fluoride (Dyneon, 99.5%), the chain transfer agent 1,4−diiodooctafluorobutane, the emulsifier ammonium 4,8-dioxa-3*H*-perfluorononanoate (ADONA, Dyneon, Gendorf, Germany), and the initiator ammonium peroxydisulfate (APS) (≥98.0%, Fluka) were used as received. Ultrapure and deionized water (electric conductivity: 0.055 µS∙cm^−1^) served as continuous phase for the emulsion polymerization. *N*,*N*−dimethyl acetamide (DMAc) (99%, Acros, Nidderau, Germany) containing 0.1% of LiBr (≥99%, Riedel-de-Häen) is used as eluent for size-exclusion chromatography. *N*,*N*−dimethylformamide−*d*_7_ (99.5%, Deutero GmbH, Kastellaun, Germany) and acetone−*d*_6_ (99.8%, Deutero GmbH, Kastellaun, Germany) were used as received.

### 2.2. Characterization

The molar mass distributions of the polymers are analyzed via size-exclusion chromatography (SEC) at a column temperature of 45 °C using DMAc which contains 0.1% LiBr as eluent. An Agilent 1200 isocratic pump, an Agilent 1200 refractive index detector, and four PSS GRAM columns (Guard, 100, 3000 and 3000 Å) from Polymer Standard Service (PSS) are used for SEC analyses at a flow rate of 1 mL∙min^−1^. Polystyrene standards (PSS) were used for calibration. Absolute molar masses were determined according to the principle of universal calibration [33]. The following Mark-Houwink parameters were used: *a*(PS) = 0.69; *K*(PS) = 0.013 mL·g^−1^; *a*(PVDF) = 0.68 und *K*(PVDF) = 0.018 mL·g^−1^. For further details the reader is referred to reference [34].^1^H NMR spectra and ^19^F NMR spectra of the polymers were recorded on a Bruker AVANCE 400 MHz spectrometer at room temperature. Acetone−*d*_6_ and *N*,*N*−dimethylformamide−*d*_7_ were used as solvents.

### 2.3. Semi-Batch Emulsion Polymerization Set-Up

The polymerizations are carried out in a 2-L reactor equipped with pressure and temperature control, agitator, baffle, rupture disc and a mass flow controller (MFC) calibrated for VDF. Details of the apparatus were given elsewhere [32]. The polymerizations are carried out at constant pressure in semi-batch mode because of the low vinylidene fluoride solubility in the aqueous phase.

### 2.4. Typical Semi-Batch Emulsion Polymerization

Deionized water is boiled under a constant nitrogen flow in order to reduce the amount of oxygen dissolved in the water. Then, the water is introduced into the pre-heated reactor at 75 °C. To further lower the oxygen content the reactor is evacuated followed by purging with nitrogen four times. While stirring the monomer VDF is added with a mass flow of 60 g·h^−1^ until the desired reaction pressure between 10 and 30 bar is reached. Via the sluice the surfactant ADONA and the chain transfer agent (CTA) I–C_4_F_8_–I is added. After stirring for 10 min in all cases 3.33 mmol of the initiator APS dissolved in 10 mL of water are added to start the polymerization. During polymerization a stirrer speed of 250 rpm is applied. The bottom opening is used to take samples to determine the solid amount gravimetrically and to evaluate the corresponding molar mass distribution of the polymer. Special care was taken to sample at the same VDF amounts fed (indicated by the MFC) to the system in all polymerizations to facilitate comparability of the data. At the end of the reaction the reactor is cooled while the pressure is slowly reduced with 0.5 bar·min^−1^ to ambient pressure. The slow expansion is required to avoid foam formation. To break the emulsion and to separate the polymer the emulsion is frozen. Then, PVDF is freed from water via filtration. Residual water is removed in vacuum at 75 °C. Table 1 summarizes the reaction conditions for all samples. Note, *n*_CTA_^in^ refers to the amount of CTA added to the reactor. *n*_CTA_^0^ gives the amount of CTA being available for chain transfer at the beginning of the reaction. As will be explained below the CTA may not only serve as chain transfer agent but may also contribute to the stabilization of the particles.

### 2.5. Kinetic Monte Carlo Modeling Approach

The simulations were executed on a workstation computer DELL Precision Tower 3620 (Intel Core i7-6700 CPU, 3.4 GHz) running a Microsoft Windows 10 Enterprise operating system. Compilation of the simulation program written in C++ was executed with Microsoft Visual Studio Community 2017. In order to generate pseudo random numbers the C++ implemented Mersenne-Twister library [35,36] was integrated into the simulation program.

## 3. Results and Discussion

### 3.1. Experimental Results

**Figure 1:**
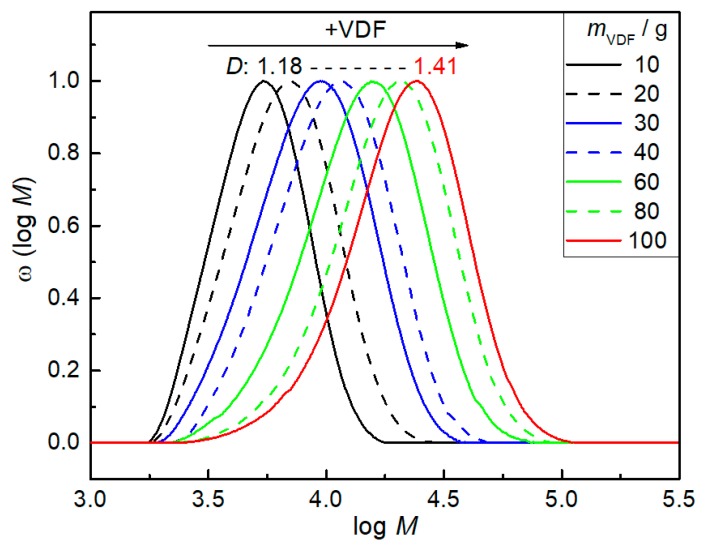
Molar mass distributions of PVDF sampled at the indicated masses of VDF introduced into the reactor. The dispersity increases from 1.18 to 1.41 for polymerizations at 75 °C and 15 bar with 7.5 mmol C_4_F_8_I_2_ (sample 7).

The semi-batch polymerizations were carried out at a constant pressure ranging from 10 to 30 bar and at a temperature of 75 °C. Due to the continuous addition of monomer the progress of the reaction is conveniently described by the mass of VDF introduced to the polymerization reactor (given by the mass flow controller) rather than by the reaction time or monomer conversion. To evaluate the variation of the molar mass distributions throughout the reaction samples of a volume of 15 mL were taken. The polymers were analyzed via SEC. As an example, Figure 1 shows the MMDs obtained from a polymerization at 15 bar and 75 °C with 7.5 mmol C_4_F_8_I_2_. The MMDs are shifted to higher molar masses with increasing amount of VDF fed to the reactor. The dispersities range from 1.18 to 1.41 for 10 and 100 g VDF polymerized, respectively, indicating very good control of the molar masses.

**Figure 2:**
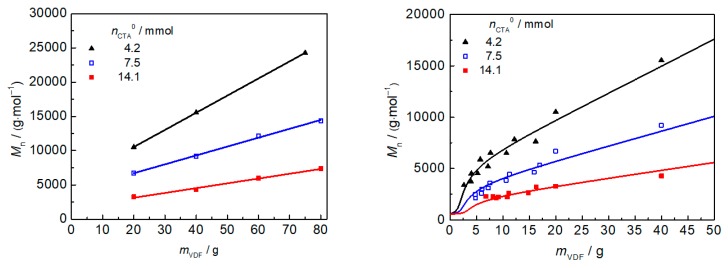
Evolution of number average molar masses with mass of VDF consumed for reactions at 75 °C and 20 bar with C_4_F_8_I_2_ amounts as indicated. *n*_CTA_^0^ is the effective amount of CTA available for chain transfer.

If the number average molar masses (*M*_n_) are considered the picture is not as ideal, as shown on the left-hand side in Figure 2 for polymerizations carried out at 75 °C and 20 bar for three CTA concentrations indicated. While *M*_n_ increases almost linearly with the VDF mass consumed, at all three CTA concentrations, extrapolation of the linear region to low VDF masses does not lead to a line crossing the ordinate at a value representing the molar mass of the initial CTA. As previously reported for VDF copolymerization controlled by C_6_F_13_I this finding may be due to non-ideality in the very early stages of the polymerization, e.g., in the initialization phase of the CTA [37]. To get a better understanding of the initial polymerization stage additional experiments (samples 4 to 6) were carried out with sampling at very low VDF masses fed into the system. The data are presented on the right-hand side of Figure 2. The data indicate that at 10 g of VDF fed to the reactor the *M*_n_ data coincide with the data at higher VDF masses. The curves were obtained from kMC modeling described in Section 3.2.

Theoretically, *M*_n_ is calculated as a function of the initial amount of CTA (*n*_CTA_^0^) and the amount of VDF polymerized (*n*_VDF_ or *m*_VDF_) in a semi-batch polymerization according to Equation (1):

(1) $$M_{n}=\frac{n_{\mathrm{VDF}}}{n_{\mathrm{CTA}}{}^{0}}\cdot M_{\mathrm{VDF}}+M_{\mathrm{CTA}}$$

In the special case of a VDF emulsion polymerization the CTA may not only serve as a transfer agent inside the particle. In fact, due to a partition equilibrium small amounts of the fluorinated CTA may also be found in the aqueous phase and on the surface of the particles, contributing to the stabilization of the particles [32]. For example, for a polymerization system particle diameters of 20 nm were estimated via dynamic light scattering after sampling. If a CTA is used, the particle diameter increased to 40 nm, suggesting that the CTA is also participating in the stabilization of the particles allowing for larger sizes. Thus, only a fraction of the CTA introduced into the reactor is actually available for the chain transfer reaction. The amount of CTA available inside the particle was estimated from the slopes of linear fits to the experimental data on the left-hand side of Figure 2. The effective CTA concentrations are 4.2 mmol, 7.5 mmol, and 14.1 mmol. These quantities are considered to be *n*_CTA_^0^. On average 57% of the CTA are available for chain transfer reactions. The low dispersity values and the linear increase in *M*_n_ with the mass of VDF fed to the system throughout most of the reaction indicate that the concentration of effective CTA does not change to a large extent during the polymerization.

**Figure 3:**
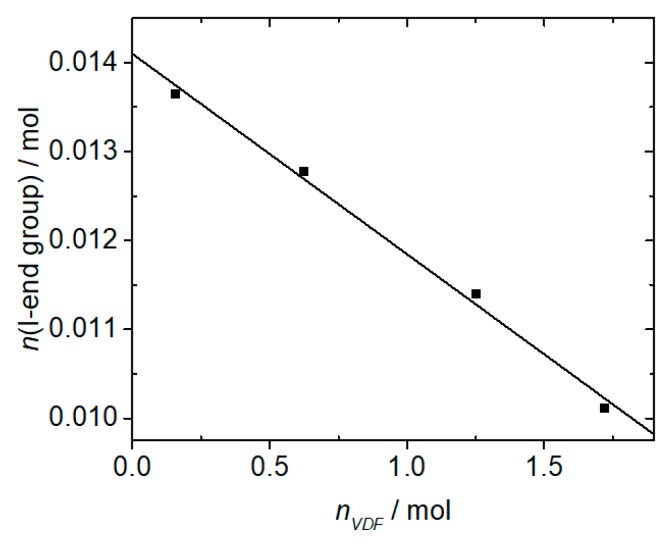
Evolution of I end group functionality with amount of VDF consumed in the semi-batch polymerization at 75 °C and 20 bar with 7.5 mmol C_4_F_8_I_2_ (sample 2).

Ideally, the number of I polymer end groups in ITP stays constant. However, it is well known that the number of I end groups is gradually decreasing with increasing degree of polymerization [38,39]. The loss of I end groups depends on the polymerization conditions. Thus, it is important to study how the I end group functionality varies throughout the emulsion polymerizations considered in this contribution. As an example, Figure 3 shows how the amount I end groups is varying with the amount of VDF polymerized. The amount and type of I end group functionality of PVDF chains is accessible from ^1^H NMR spectroscopy. As previously detailed due to head to tail, tail to tail, and head to head addition of the monomer VDF to the propagating chain two types of iodine end groups are found in ITP [10]: –CH_2_–CF_2_–I and –CF_2_–CH_2_–I. The protons in both types of end groups are associated with ^1^H NMR signals at 3.71 ppm and 3.97 ppm [10]. For PVDF obtained from a reaction at 75 °C and 20 bar with 7.5 mmol CTA 93% of iodine end groups are –CF_2_–CH_2_–I. The finding of an increasing fraction of CF_2_–CH_2_–I end group with an increase in chain length is in good agreement with previous work, e.g., by the group of Ameduri [11,40]. Using C_6_F_13_I as CTA the fraction of CF_2_–CH_2_–I end groups increased from 25% at a chain length of 6 to 72% for a chain length of 25. The emulsion polymerization at 75 °C and 20 bar with 7.5 mmol C_4_F_8_I_2_ results in 96% of the CH_2_–I end group already at a degree of polymerization of 88, which is associated with the data point at the lowest amount of VDF polymerized in Figure 3.

Figure 3 clearly shows a linear decrease of I end group functionality during the course of the polymerization. The more VDF is polymerized the lower the amount of I end groups. The reason for this lowering is suggested to be due to an irreversible deactivation of the I end groups via H transfer from initiator or surfactant as well as via bimolecular termination. This type of deactivation was already reported for RAFT VDF polymerizations [6]. Since both RAFT and ITP are considered to be polymerizations with degenerative transfer, the linear correlation between the decreasing amount of I end groups and the amount of VDF consumed may be interpreted as a measure for the irreversible deactivation of I polymer end groups or in other words the loss of I end groups. The inverse of the slope of the linear fit in Figure 3 yields the average number of propagation steps after which one of the above-listed deactivation reactions occurs. The diagram allows for identification of the proportionality of two reaction pathways: deactivation and propagation. Transfer reactions do not impact the graph in Figure 3. The linear fit of the data is associated with an absolute value of 0.00225 for the slope, which may be interpreted as the probability that irreversible deactivation of an I end group occurs at the radical chain end rather than addition of a monomer unit in a propagation reaction. The linear fit suggests an initial value of the molar amount of I end groups of 14.1 mmol, which is slightly lower than the theoretical value of 15 mmol for *n*_CTA_^0^ = 7.5 mmol of sample 2 in Table 1. The reason may be seen in a degradation of the CTA agent during storage. In the kinetic modeling described below the concentration of active CTA molecules present in the particles was considered.

### 3.2. Kinetic Monte Carlo Simulation

In order to get a better understanding of the ITP process—in particular in the initial phase of the reaction—the deactivation–activation equilibrium of the initial CTA and the polymeric species is investigated via kinetic Monte Carlo (kMC) modeling of the polymerization inside the particles. In case of a monofunctional CTA the following equilibria are considered:

(2) $$P_{m}^{•}+I-X⇌P_{m}-I+X^{•}$$

(3) $$P_{n}^{•}+P_{m}-I⇌P_{n}-I+P_{m}^{•}$$

The first equilibrium describes the transformation of the initial CTA I–X via reaction with a radical of chain length m. The radical, X^•^, initiates a new chain leading to a polymeric radical. This process is frequently referred to as the initialization period. Equation (3) describes the main equilibrium of the ITP process. For details on ITP the reader is referred to the literature [10,11,38]. Equations (2) and (3) refer to the use of a CTA with a single iodine atom, as for example in C_6_F_13_I (X = C_6_F_13_). In the case of bifunctional CTAs with two iodine end groups the reaction mechanism is more complex. In this contribution I–X–I with X = C_4_F_8_ is considered. As shown in Equations (4) and (5) firstly one of the I atoms undergoes a chain transfer reaction to yield I–X followed by addition of monomer to this radical. After transfer with an I atom a mixed intermediate I–XP_n_–I is formed. Equations (6) and (7) illustrate the activation of the second I atom of the original CTA resulting in I-P_n_X^•^ and subsequent propagation yielding a radical with two polymeric segments, I–P_n_XP_k_^•^. Finally, in Equation 8 the main equilibrium between species containing two polymeric segments is shown.

A clear distinction between pre-equilibrium and main equilibrium is not feasible. For example, the intermediate species I–XP_n_–I contains an I atom in the neighborhood of X, the fluorinated fragment of the initial CTA, and a second I next to a polymer segment. Thus, I–XP_n_–I may undergo two different chain transfer reactions. Equations (4)–(8) represent only a small selection of reactions occurring during ITP with C_4_F_8_I_2_. For example, instead of P_m_^•^ in Equations (4) and (6) the radicals I–XP_n_^•^ or I–P_n_XP_k_^•^ may be in equilibrium with I–X–I or I–XP_n_–I, respectively. The full set of 63 reactions occurring is given in the Supplementary Materials.

(4) $$P_{m}^{•}+I-X-I⇌P_{m}-I+I-X^{•}$$

(5) $$I-X^{•}+\mathrm{nM}\to I-{\mathrm{XP}}_{n}^{•}$$

(6) $$P_{m}^{•}+I-{\mathrm{XP}}_{n}-I⇌P_{m}-I+I-P_{n}X^{•}$$

(7) $$I-P_{n}X^{•}+\mathrm{kM}\to I-P_{n}{\mathrm{XP}}_{k}^{•}$$

(8) $$I-P_{n}{\mathrm{XP}}_{m}^{•}+I-P_{k}{\mathrm{XP}}_{1}-I⇌I-P_{n}{\mathrm{XP}}_{m}-I+I-P_{k}{\mathrm{XP}}_{1}^{•}$$

The kMC simulation accounts for a large ensemble of discrete molecules. The number of molecules for all reactants of the model is proportional to the number of each species inside the latex particles. The molar amount of the gaseous monomer VDF inside the particles depends on the pressure inside the reactor and may be considered constant due to the semi-batch mode. To estimate this quantity experimentally the reactor filled with water and surfactant was pressurized with 20 bar of VDF for one hour to dissolve VDF in the micelles. Then, the reactor was slowly depressurized and VDF dissolved inside the particles was polymerized at ambient pressure. The reaction resulted in 2 g of PVDF, which is associated with the consumption of 31.2 mmol VDF.

At the beginning of the polymerization, bifunctional CTA is contained in the micelles. The model accounted for 6% of the iodine atoms being already deactivated (end group D instead of I) due to aging, which was identified via ^19^F NMR spectroscopy. For this purpose in addition to the CTA I–X–I a CTA with one deactivated group D–X–I is introduced in the simulation. According to the molar amounts of VDF, I–X–I and D–X–I these species are distributed to 10^7^ molecules. Contrary to a kinetic Monte Carlo simulation of an emulsion polymerization with discrete particles and a corresponding control volume [20], in the simulation described here only the propagation and transfer reactions inside the particles are considered. The reactions inside the water phase and the radical transport into the particle are not part of the model. This approach is justified since due to the semi-batch process the molar amount of monomer (*n*_M_) inside the particle is constant. Consequently, the number of VDF molecules in the simulation is also constant. According to the ITP mechanism, the CTA molecules and the resulting polymer species with I end groups control the radical polymerization inside the particles. It may be assumed that the transfer and propagation reactions inside the particles are dominant due to the comparably high CTA concentration. Thus, the molar amount of dead polymer molecules generated due to bimolecular termination is negligible compared to the amount of propagating chains. The experimentally observed linear increase in *M*_n_ as a function of the polymerized mass of VDF and low dispersities support this assumption. Therefore, the termination reactions are not contained in the kinetic model and *M*_n_ is simulated on the basis of propagation and transfer reactions.

Consideration of all the reactions mentioned the species listed in Table 2 have to be accounted for. For the macromolecular species #2, #3, #5 to #9, in addition to the number of molecules the chain lengths of each molecule is registered in the simulation. The simulation starts in a first Monte Carlo step with the reaction of a radical of type I–X^•^, which is formed upon activation of a CTA molecule I-X-I. The equations for the probabilities of individual transfer reactions contained in Table 2 are given by the reaction rate of this individual transfer reaction divided by the reaction rates of all other reactions: propagation and transfer reactions of I–X and I–P. In addition, the individual rate coefficients *k*_p_, *k*_tr_ und *k*_ex_ were substituted by *C*_tr_ and *C*_ex_.

**Scheme 1:**
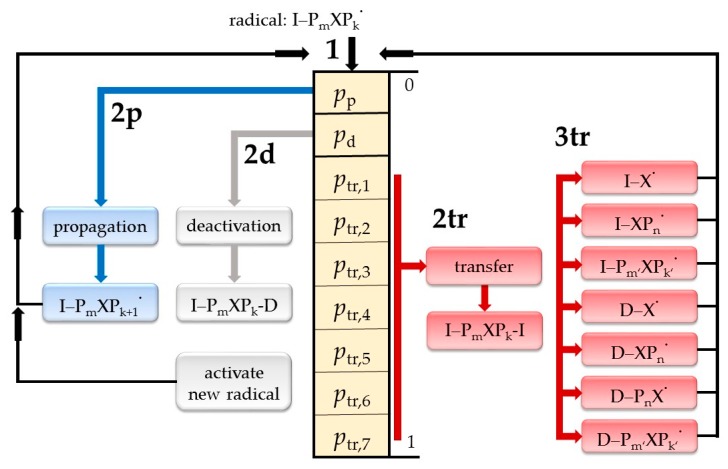
Illustration of a Monte Carlo step for the reaction of a radical of type I–P_m_XP_k_^•^.

A single Monte Carlo step in the kMC simulations accounts for the execution of a reaction according to the actual reaction probabilities. As an example for a Monte Carlo step Scheme 1 depicts the reactions that may occur with a radical of type I–P_m_XP_k_^•^. The radical I–P_m_XP_k_^•^ (i) may add a monomer unit in a propagation reaction leading to the radical I–P_m_XP_k+1_^•^, (ii) may be irreversibly deactivated resulting in species I–P_m_XP_k_–D, (iii) or may be reversibly deactivated due to a transfer reaction with an I end group of another molecule resulting in I–P_m_XP_k_–I. The actual reaction path is selected in step 1 of Scheme 1 using a random number r being in the interval 0 ≤ r ≤ 1. The random number r is a pseudo random number, which is generated using the Mersenne-Twister algorithm [35]. The probability of the propagation reaction *p*_p_ is calculated according to Equation (9) using the molar amount, *n*_M_, of the monomer VDF, the molar amount, Σ*n*_X-I_, of the I end groups of the original CTA (I–X–I) or the partially initialized CTA (I–XP_n_–I), and the molar amount, Σ*n*_P-I_, of the I end groups of the PVDF segments. It is important to note that the type of I end groups is different: CF_2_–I is the I end group in the original CTA, whereas –CH_2_–I is the predominant I end group of the PVDF segment. The transfer activities of both chain ends are expected to differ significantly [10] and, thus, within the ITP mechanism two different transfer constants, *C*_tr_ and *C*_ex_, are defined by Equations (11) and (12), respectively. *C*_tr_ refers to the transfer reaction of –CF_2_–I and *C*_ex_ to the transfer reaction of the –CH_2_–I end group. Both transfer constants were determined by a fit of the simulation to the experimental data shown in Figure 2 assuming that the transfer constants are not significantly affected by the slightly different reaction pressure.

(9) $$p_{p}=\frac{n_{M}}{n_{M}+C_{\mathrm{tr}}\cdot {\sum n}_{X-I}+C_{\mathrm{ex}}\cdot {\sum n}_{P-I}}-p_{d}$$

(10) $$p_{d}=0.00225\cdot p_{p}$$

(11) $$C_{\mathrm{tr}}=\frac{k_{\mathrm{tr},X-I}}{k_{p}}$$

(12) $$C_{\mathrm{ex}}=\frac{k_{\mathrm{tr},P-I}}{k_{p}}$$

(13) $$p_{p}+p_{d}+\sum_{i=1}^{7}p_{\mathrm{tr},i}=1$$

The overall probability of all reactions in an MC step equals one and is given by Equation (13). It is calculated as the sum of *p*_p_, the probability for irreversible deactivation *p*_d_, and the sum of probabilities of the transfer reactions of the seven I end groups containing species accounted for in the mechanism. The transfer probabilities *p*_tr,I_ (I = 1 to 7) of species #1 to #7 are listed in Table 2. For all species the number and type of end groups is considered. Therefore, the transfer probability of the deactivated species #8 and #9, which contain no I, is zero. Since the random number r and the probability vector in Scheme 1 (the sum of *p*_p_ to *p*_tr,7_ in Equation (13) are in the value range from zero to one, the random number r allows for an easy selection of a reaction path. Starting with sector *p*_p_ it is tested, to which sector r belongs.

If the random number generated in step 1 of Scheme 1 is r ≤ *p*_P_, transfer reactions do not take place and the simulation reaches stage 2p in the next step. The notation 2p refers to step 2 and the propagation path. In case of a propagation reaction the length of the macroradical is increased by one (I–P_m_XP_k+1_^•^) and the radical is transferred to the next MC step of the simulation.

If the random number fulfills *p*_p_ < r ≤ (*p*_p_ + *p*_d_) an irreversible deactivation reaction occurs at the chain end (2d). A molecule of type I–P_m_XP_k_–D is generated and a new MC step is started. To provide a new radical for the execution of the next MC step, a molecule is selected from the reservoir of reversibly deactivated species (#1 to #7 in Table 2) according to statistical weighting based on the actual number of molecules and the number of I end groups (1 and 2 in Table 2) with a random number. The selected molecule is activated by taking off the I end group. Subsequently, the radical keeping its chain length is introduced into the next MC step.

In case of the random number being r > (*p*_p_ + *p*_d_) in the first step (2tr) a transfer reaction is carried out. The initially growing radical is reversibly deactivated and is stored with its chain length as a species of type I–P_m_XP_k_–I. The reaction partner is determined in step 3tr with the random number r and the transfer probabilities *p*_tr,1_ to *p*_tr,7_. For example, transfer with species #2 (I–XP_n_–I) occurs, which is activated by removal of an I end group, if r is in the interval (*p*_p_ + *p*_d_ + *p*_tr,1_) < r ≤ (*p*_p_ + *p*_d_ + *p*_tr,1_ + *p*_tr,2_). The radical generated is then used in the next MC step. If transfer to a low molar mass species (I–X–I or D–X–I) occurs, in the next MC step a new propagating chain is generated.

The results are logged at fixed intervals of VDF consumed; e.g., a simulation run up to a conversion of 120 g VDF and data export every 0.05 g affords a simulation time of 18 min. If data are logged only every 1 g of consumed VDF the simulation runs only for 1 min. This finding indicates that the time-determining step is the analysis of the chain length distributions required for the calculation of *M*_n_. The MC steps are not rate determining for the simulation. Within a Monte Carlo simulation the system size is an important parameter with respect to obtaining reproducible results. Here, the number of molecules used is 10^7^, which represents the molar ratio of monomer and CTA at the beginning of the simulation. This number was selected to be sufficiently large to generate reproducible results with a moderate demand of computing time. Averaging of the simulation runs is not required. Even an enhancement of the molecular number from 10^7^ to 10^8^ does not lead to significant variations in the simulation results.

**Figure 4:**
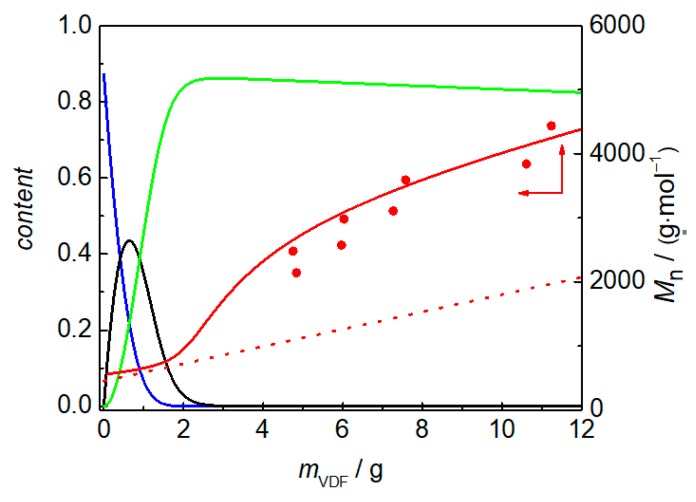
Evolution of the original CTA (blue), the partially transformed CTA (black), fully transformed CTA (green) and number average molar mass (red) during the early stages of the polymerization. The experimental points (red dots) and modelled number average molar masses refer to a reaction at 20 bar with 7.5 mmol I–C_4_F_8_–I (sample 5).

**Figure 5:**
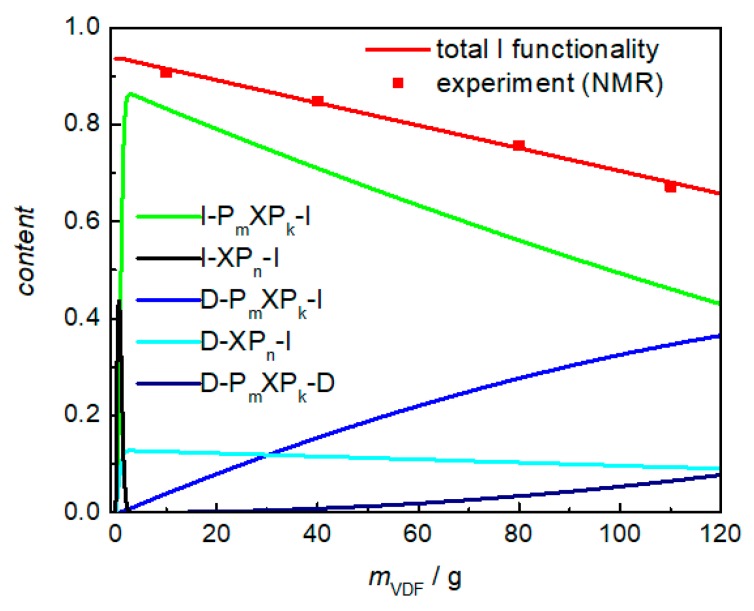
Evolution of the indicated polymeric iodine containing species during the VDF emulsion polymerization. In addition, non-functionalized species D–P_m_–R–P_k_–D are contained (sample 2).

The kMC modeling yields the variation of all deactivated species during the polymerization. In the initial phase of the reaction the original CTA (I–X–I), the partially transformed CTA (I–XP_n_–I) and the fully transformed CTA species (I–P_m_XP_k_–I) are of particular interest. The disappearance of the original CTA and the appearance of the fully transformed species indicates how quickly the main equilibrium required for molar mass control during the polymerization is established. The data are shown in Figure 4. It is clearly seen that the original CTA is fully consumed at a VDF consumption of 2 g. Considering a total VDF consumption of 110 g at the end of the reaction the transformation may be considered very fast. The mixed species I–XP_n_–I given in black is consumed after consumption of 4 g of VDF. At this point the bifunctional deactivated polymeric species reaches its maximum followed by a slight decrease due to irreversible deactivation. Further, the red data in Figure 4 refer to the evolution of the number average molar masses for an ideal ITP (dashed) as well as the experimentally derived and modeled data. The polymeric species occurring throughout the entire reaction are depicted in Figure 5. As seen in Figure 5, the maximum content of the bifunctional deactivated polymeric species (I–P_m_XP_k_–I) is quickly formed followed by a steady decay due to irreversible deactivation reactions. After 120 g of VDF are consumed, around 40% of the species contain two iodine end groups. The fraction of the fully transformed species with only a single iodine atom (D–P_m_XP_k_–I) is gradually increasing until a value of almost 40% is reached at the end of the simulation. Moreover, around 10% of the species resemble irreversible deactivation of one side of the original CTA during the initialization of the original CTA (D–XP_n_–I). The figure clearly indicates that the fraction of polymeric species without an iodine atom is rather small. At the end of the simulation around 10% of the species are irreversibly deactivated. The red data refer to the overall functionality; that is, the sum of mono- and bifunctional deactivated species. It is clearly shown that around 70% of the initially present iodine end groups are present at the end of the reaction. Moreover, excellent agreement between experimental simulation results is reached.

### 3.3. Discussion

The simulations result in *C*_tr_ = 7.1 and *C*_ex_ = 0.094 by fitting the experimental data in Figure 2. Figure S1 of the Supplementary Material gives the joint confidence intervals for the parameters and Figure S2 demonstrates the sensitivity of the parameters. These data are compared with literature values for ITP and RAFT polymerization listed in Table 3. Boyer et al. reported transfer constants for the –CF_2_–I end group in VDF telomerizations ranging from 7.4 to 7.9 [10]. These values are in very good agreement with the value of 7.1 for *C*_tr_ determined in this contribution and lie within the confidence interval depicted in Figure S1. In addition, a transfer constant of 0.3 was estimated by Boyer et al. [10] for a model compound with –CH_2_–I end group. This value is significantly higher than the value for *C*_ex_ determined in this contribution. In addition, it was stated that transfer constants higher than one are required for a well-controlled polymerization [10]. In the following it is explained why good control of the polymerization up to high degrees of polymerization of around 400 was still achieved.

In Figure 4 it is seen that after polymerization of only 10 g VDF the transformation of –CF_2_–I to –CH_2_–I is complete. Thus, from this stage on only end groups with the lower transfer constant of 0.094 are available for iodine transfer. In order to achieve good control in reversible deactivation transfer polymerizations, the probability for reversible transfer at the chain end has to be high compared to the probability of the propagation reaction. This is reflected in the definition of the transfer constant in Equation (12) as the ratio of the rate coefficients for the transfer and propagation reaction. Since both reactions are of second order, additionally, the ratio of the initial concentrations of monomer and CTA, *c*_mon_^0^/*c*_CTA_^0^, determines the reaction path. Thus, an evaluation of the transfer constants has to be accompanied by a consideration of the concentrations present. For this task the transfer probability, *p*_transfer_, given in Equation (14) may be calculated. In the case of a bifunctional CTA, such as C_4_F_8_I_2_, c_CTA_^0^ in Equation (14) has to be doubled. The use of *p*_transfer_ is rather convenient since only the initial concentrations and the transfer constants are required. The transfer probabilities, *p*_tr_, introduced above in the section on kMC simulations refer to the situation at a certain instant throughout the polymerization with momentary concentrations or molar amounts of individual species. Here, the notation *C*_transfer_ is used to distinguish that literature data generally refer to the overall transfer activity in ITP and RAFT polymerizations.

(14) $$p_{\mathrm{transfer}}=\frac{C_{\mathrm{transfer}}\cdot c_{\mathrm{CTA}}{}^{0}/c_{\mathrm{mon}}{}^{0}}{1+C_{\mathrm{transfer}}\cdot c_{\mathrm{CTA}}{}^{0}/c_{\mathrm{mon}}{}^{0}}$$

The values for *p*_transfer_ in Table 3 were calculated according to the information in the original publications. It is clearly seen that *p*_transfer_ values for ITP with –CF_2_–I end groups (entries #1 and #2) are at least one order of magnitude higher than the values for the RAFT systems. These high probabilities are due to rather small ratios of *c*_mon_^0^/*c*_CTA_^0^, which limit the accessible number average degrees of polymerization, *DP*_n_, at complete conversion in batch polymerizations. With *c*_mon_^0^/*c*_CTA_^0^ = 100/6.6 (entry #1 in Table 3), a maximum value of *DP*_n_ = 15 may be reached. On the contrary in semi-batch polymerizations this limit is not operative since monomer is continuously fed to the system. Despite a rather low transfer constant of 0.094 for ITP with –CH_2_–I end groups a rather high transfer probability of 0.043 is reached in semi-batch polymerization mode. For comparison, ITP of VDF with HCF_2_CF_2_CH_2_–I is associated with a transfer probability of 0.019. To judge these transfer probabilities it is instructive to consider more common systems, such as RAFT polymerizations of styrene. For example, Ran et al. reported transfer constants for trithiocarbonates as RAFT agents ranging from 4.32 to 23.2 [42]. Two examples are given in Table 3 (entries #7 and #8). While insufficient control was achieved for #8 with *C*_transfer_ = 4.32 and *p*_transfer_ = 0.009, system #7 with *C*_transfer_ = 23.2 and *p*_transfer_ = 0.044 is very well-controlled. The ratio of concentrations *c*_mon_^0^/*c*_CTA_^0^ = 100/0.2 is associated with a maximum *DP*_n_ of 500. The comparison indicates that despite a low transfer constant of 0.094 the use of a bifunctional CTA in semi-batch ITP of VDF yields a similar *p*_transfer_ of 0.043 compared to 0.044 in the well-controlled styrene RAFT polymerizations. These values are significantly higher than *p*_transfer_ = 0.007 for ITP of styrene, and clarifies why this system is not very well controlled. This discussion explains why semi-batch emulsion ITP of VDF may be carried out and well controlled up to rather high *DP*_n_ despite the low transfer constant.

**Figure 6:**
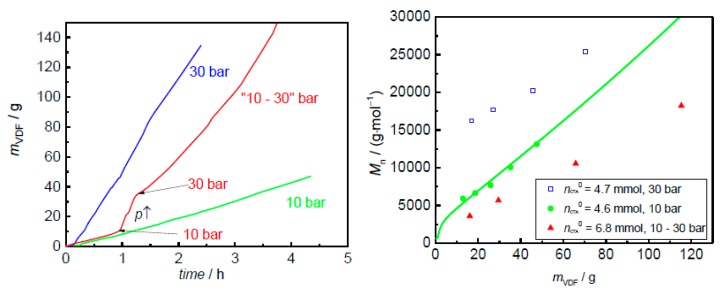
VDF mass fed to the reactor as a function of time during the VDF emulsion polymerization (sample 8–10) at the indicated pressures (**left**) and evolution of number average molar masses, *M*_n_, with VDF mass consumed for reactions at 10, 30 bar, and a pressure profile (**right**). The line in the right diagram was obtained via modeling. For further details the reader is referred to the text.

In semi-batch emulsion polymerizations of VDF the pressure controls the monomer concentration inside the particles. According to Henry’s law, the molar amount of VDF increases from *n*_VDF_^0^ = 15.6 mmol at 10 bar, to 31.2 mmol at 20 bar, and to 46.8 mmol at 30 bar. As shown on the left-hand side of Figure 6, the variation of pressure is associated with a significant increase in rate of polymerization. While at 30 bar 100 g PVDF are obtained after 2 h, at 10 bar only 40 g PVDF are obtained in 4 h. These numbers refer to polymerization rates of 0.78 mol·h^–1^ and 0.16 mol·h^−1^ at 30 and 10 bar, respectively. The impact of pressure on *M*_n_ is depicted on the right-hand side of Figure 6 at 10 bar a steady increase in *M*_n_ with mass of consumed VDF is observed. At 30 bar, at the beginning of the reaction rather high *M*_n_ values of 16,000 g∙mol^−1^ are reached. With further polymerization this value gradually increases. As discussed above, the rather high *M*_n_ value at the beginning of the reaction is due to the fact that the transformation of the original CTA C_4_F_8_I_2_ is slow compared to the propagation rate. While the equilibria of the various species with I end groups have to be established until only polymeric species with I end groups are present (in particular, at 30 bar), significant propagation occurs due to the higher VDF content in the particles. At 10 bar, the equilibria also have to be established. However, at 10 bar, the amount of VDF inside the particles is significantly lower, thus, less propagation occurs during this stage of the polymerization, in which the system is not yet well controlled. If *p*_transfer_ listed in Table 4 is considered, the lower molar amount of VDF directly leads to a higher transfer probability. If the molar amount of VDF is increased the transfer probability is reduced. Of course, *p*_transfer_ may also be increased due to an increase in CTA concentration.

Based on this discussion a polymerization process was inferred that allows for high polymerization rates at high pressure and, at the same time, well-controlled molar mass distributions are obtained up to high degrees of polymerization. For this purpose, a pressure profile was used: in the initial phase of the polymerization a low pressure of 10 bar was chosen to reach a high transfer probability. Once the main equilibrium is established the pressure is increased to 30 bar to take advantage of the higher monomer content in the particles and the associated higher polymerization rate. To further enhance the transfer probability, the CTA amount was increased to 6.8 mmol. Thus, in the initial phase of the polymerization, *p*_transfer_ of 0.861 is reached leading to better control of the activation of the CTA. Later, after increasing the pressure to 30 bar, *p*_transfer_ is reduced to 0.026.

The evolution of VDF consumed with time is depicted on the left-hand side of Figure 6. For the first hour, a pressure of 10 bar is chosen, which leads to a consumption of 10 g of VDF. Then, within the next 20 min, the pressure is slowly increased to 30 bar. When the final pressure is reached around 35 g of VDF are consumed. In the following period, the consumption of VDF with time is very similar to the polymerization carried out at 30 bar from the beginning. The number average molar masses given on the right-hand side of Figure 6 indicate that a slower increase of *M*_n_ with amount of VDF consumed is found. The data indicate that the low transfer probability of 0.026 is sufficient to keep the well-controlled polymerization conditions established in the initial phase.

## 4. Conclusions

Semi-batch emulsion polymerizations of VDF were performed at 75 °C and pressures ranging from 10 to 30 bar. Well-controlled reaction conditions are achieved for the iodine transfer polymerization using C_4_F_8_I_2_ as chain transfer agent. The experiments are accompanied by kinetic Monte Carlo simulations of the reversible deactivation transfer process. Number average molar masses are obtained as a function of VDF fed to the system. In addition, kMC modeling provides access to the concentrations of all species occurring during the semi-batch polymerization and a detailed understanding of the underlying transfer process is reached. Based on the modeling results, the non-ideal increase of *M*_n_ frequently observed in the initial phase of the process and being particularly pronounced at high pressure and, consequently, at high monomer concentration, may be understood.

Chain transfer constants for the original CTA and for the dominant –CH_2_–I end group of the polymeric species are estimated to be 7.1 and 0.094, respectively. Despite the fact that the transfer constant for the polymeric I end group is rather low compared to transfer constants in RAFT polymerization system, the system is very well controlled, because the ratio of monomer and CTA concentration is high due to the semi-batch mode. Upon variation of reaction pressure this ratio may dynamically be tuned. Based on the modeling results, an optimized polymerization process is inferred. In the crucial activation phase of the bifunctional CTA, a rather low pressure of 10 bar is selected to limit the propagation events while the activation-deactivation equilibria are established. Then, the pressure is increased to 30 bar to enhance monomer concentration inside the particles and reach higher polymerization rates. It is confirmed experimentally that this two-pressure process is associated with excellent molar mass control at high polymerization rate.

## Figures and Tables

**Table 1:** Reaction conditions for polymerizations carried out at 75 °C: (a) amount of CTA added to the reactor and (b) amount of CTA available for chain transfer.

Sample	*ρ*/Bar	*n*_CTA_^in^_/_mmol ^(a)^	*n*_CTA_^0^_/_mmol ^(b)^
1, 4	20	6.7	4.2
2, 5	20	13.4	7.5
3, 6	20	26.8	14.1
7	15	13.4	7.5
8	10	6.7	4.6
9	30	6.7	4.7
10	10–30	13.4	6.8

**Table 2:** Species accounted for in the kMC model. X: C_4_F_8_; P_n_, P_m_ and P_k_ refer to polymeric chains of chain length n, m and k, respectively. D indicates irreversibly deactivated chain ends.

I end Groups	Species Name	Number	Transfer Probability
2	I–X–I	#1	$p_{\mathrm{tr},1}=\frac{C_{\mathrm{tr}}\cdot 2\cdot n_{\mathrm{I-X-I}}}{n_{M}+C_{\mathrm{tr}}\cdot {\sum n}_{X-I}+C_{\mathrm{ex}}\cdot {\sum n}_{P-I}}$
	I–XP_n_–I	#2	$p_{\mathrm{tr},2}=\frac{C_{\mathrm{tr}}\cdot n_{I-{\mathrm{XP}}_{n}-I}+C_{\mathrm{ex}}\cdot n_{I-{\mathrm{XP}}_{n}-I}}{n_{M}+C_{\mathrm{tr}}\cdot {\sum n}_{X-I}+C_{\mathrm{ex}}\cdot {\sum n}_{P-I}}$
	I–P_m_XP_k_–I	#3	$p_{\mathrm{tr},3}=\frac{C_{\mathrm{ex}}\cdot 2\cdot n_{\mathrm{I-PmXPk-I}}}{n_{M}+C_{\mathrm{tr}}\cdot {\sum n}_{X-I}+C_{\mathrm{ex}}\cdot {\sum n}_{P-I}}$
1	D–X–I	#4	$p_{\mathrm{tr},4}=\frac{C_{\mathrm{tr}}\cdot n_{\mathrm{D-X-I}}}{n_{M}+C_{\mathrm{tr}}\cdot {\sum n}_{X-I}+C_{\mathrm{ex}}\cdot {\sum n}_{P-I}}$
	D–XP_n_–I	#5	$p_{\mathrm{tr},5}=\frac{C_{\mathrm{ex}}\cdot n_{D-{\mathrm{XP}}_{n}-I}}{n_{M}+C_{\mathrm{tr}}\cdot {\sum n}_{X-I}+C_{\mathrm{ex}}\cdot {\sum n}_{P-I}}$
	I–XP_n_–D	#6	$p_{\mathrm{tr},6}=\frac{C_{\mathrm{tr}}\cdot n_{I-{\mathrm{XP}}_{n}-D}}{n_{M}+C_{\mathrm{tr}}\cdot {\sum n}_{X-I}+C_{\mathrm{ex}}\cdot {\sum n}_{P-I}}$
	D–P_m_XP_k_–I	#7	$p_{\mathrm{tr},7}=\frac{C_{\mathrm{ex}}\cdot n_{D-P_{m}{\mathrm{XP}}_{k}-I}}{n_{M}+C_{\mathrm{tr}}\cdot {\sum n}_{X-I}+C_{\mathrm{ex}}\cdot {\sum n}_{P-I}}$
0	D–XP_n_–D	#8	$p_{\mathrm{tr},8}=0$
	D–P_m_XP_k_–D	#9	$p_{\mathrm{tr},9}=0$

**Table 3:** Comparison of transfer constants, *C*_transfer_, and transfer probabilities, *p*_transfer_, for ITP and RAFT polymerizations; CTA: chain transfer agent; *T*: temperature; 0 refers to initial concentrations. ^(^^*)^: *n*_VDF_^0^ = 31.2 mmol at 20 bar, *n*_CTA_^0^ = 7.5 mmol.

Entry	CTA	*T*/°C	Monomer	*c*_mon_^0^/*c*_CTA_^0^	*C*_transfer_	*p*_transfer_
#1	C_6_F_13_I [10]	75	VDF	100/6.6	7.9	0.343
#2	C_6_F_13_CH_2_CF_2_I [10]	75	VDF	100/6.6	7.4	0.328
#3	HCF_2_CF_2_CH_2_I [10]	75	VDF	100/6.6	0.3	0.019
#4	C_4_F_8_I_2_^this work^	75	VDF	100/24.0 ^(*)^	7.1	0.773
#5	IP_m_C_4_F_8_P_k_I ^this work^	75	VDF	100/24.0 ^(*)^	0.094	0.043
#6	polystyrene-I [41]	80	styrene	100/0.2	3.6	0.007
#7	(HO(=O)C(CH_3_)_2_S)_2_C=S [42]	70	styrene	100/0.2	23.2	0.044
#8	(HO(=O)CC_2_H_4_S)_2_C=S [42]	70	styrene	100/0.2	4.32	0.009

**Table 4:** Transfer probabilities for semi-batch iodine transfer polymerizations for variation of pressure with X = C_4_F_8_: (a) pressure during the initial phase of the polymerization; (b) pressure in the second phase of the polymerization.

CTA	*p*/bar	*n*_VDF_^0^/mmol	*n*_CTA_^0^/mmol	*c*_mon_^0^/*c*_CTA_^0^	*C*_transfer_	*p*_transfer_
I–X–I	10	15.6	4.6	100/29.5	7.1	0.807
I–P_m_XP_k_–I	10	15.6	4.6	100/29.5	0.094	0.053
I–X–I	20	31.2	4.2	100/13.4	7.1	0.656
I–P_m_XP_k_–I	20	31.2	4.2	100/13.4	0.094	0.025
I–X–I	30	46.8	4.7	100/10.0	7.1	0.587
I–P_m_XP_k_–I	30	46.8	4.7	100/10.0	0.094	0.018
I–X–I	10 ^(a)^	15.6	6.8	100/43.6	7.1	0.861
I–P_m_XP_k_–I	30 ^(b)^	46.8	6.8	100/14.5	0.094	0.026

## Supplementary Materials

The following are available online at http://www.mdpi.com/2073-4360/10/9/1008/s1, Scheme S1: Transfer and propagation reactions included in the kMC model. Figures S1 and S2: joint confidence interval and sensitivity of the parameters *C*_tr_ und *C*_ex_, respectively.
