# Supplementary material for: Kinetic Monte Carlo Simulation Based Detailed Understanding of the Transfer Processes in Semi-Batch Iodine Transfer Emulsion Polymerizations of Vinylidene Fluoride
Source: Polymers (Basel). 2018 Sep 10;10(9):1008. doi: 10.3390/polym10091008 (PMC6403726; doi:10.3390/polym10091008)
Supplement: Supplementary file 1 [file polymers-10-01008-s001.pdf]

Supplementary Material

Kinetic Monte Carlo simulation based detailed understanding of the transfer processes in semi-batch iodine transfer emulsion polymerizations of vinylidene fluoride

Florian Brandl, Marco Drache, Sabine Beuermann

Scheme S1: Transfer and propagation reactions included in the kMC model.

with I- : iodine end group;

D-: irreversibly deactivated end group

X, X‘: C_4F_8

reactions of I-X•

I-X•	+	I-X‘-I	→	I-X-I	+	I-X’•
I-X•	+	D-X‘-I	→	I-X-I	+	D-X’•
I-X•	+	I-X‘P_m-I	→	I-X-I	+	I-X’P_m•
I-X•	+	I-X‘P_m-I	→	I-X-I	+	I-P_mX’•
I-X•	+	D-X‘P_m-I	→	I-X-I	+	D-X’P_m•
I-X•	+	I-X‘P_m-D	→	I-X-I	+	D-P_mX’•
I-X•	+	D-P_mX‘P_k-I	→	I-X-I	+	D-P_mX’P_k•
I-X•	+	I-P_mX‘P_k-I	→	I-X-I	+	I-P_mX’P_k•
I-X•	+	M	→	I-XP_1•		

reactions of D-X•

D-X•	+	I-X‘-I	→	D-X-I	+	I-X’•
D-X•	+	D-X‘-I	→	D-X-I	+	D-X’•
D-X•	+	I-X‘P_m-I	→	D-X-I	+	I-X’P_m•
D-X•	+	I-X‘P_m-I	→	D-X-I	+	I- P_mX’•
D-X•	+	D-X‘P_m-I	→	D-X-I	+	D-X’P_m•
D-X•	+	I-X‘P_m-D	→	D-X-I	+	D-P_mX’•

$D-X^{\bullet} + D-P_mX'P_k-I \rightarrow D-X-I + D-P_mX'P_k^{\bullet}$

$D-X^{\bullet} + I-P_mX'P_k-I \rightarrow D-X-I + I-P_mX'P_k^{\bullet}$

$D-X^{\bullet} + M \rightarrow D-XP_1^{\bullet}$

<u>reactions of $I-XP_n^{\bullet}$</u>

$I-XP_n^{\bullet} + I-X'-I \rightarrow I-XP_n-I + I-X'^{\bullet}$

$I-XP_n^{\bullet} + D-X'-I \rightarrow I-XP_n-I + D-X'^{\bullet}$

$I-XP_n^{\bullet} + I-X'P_m-I \rightarrow I-XP_n-I + I-X'P_m^{\bullet}$

$I-XP_n^{\bullet} + I-X'P_m-I \rightarrow I-XP_n-I + I-P_mX'^{\bullet}$

$I-XP_n^{\bullet} + D-X'P_m-I \rightarrow I-X-P_n-I + D-X'P_m^{\bullet}$

$I-XP_n^{\bullet} + I-X'P_m-D \rightarrow I-X-P_n-I + D-P_mX'^{\bullet}$

$I-XP_n^{\bullet} + D-P_mX'P_k-I \rightarrow I-X-P_n-I + D-P_mX'P_k^{\bullet}$

$I-XP_n^{\bullet} + I-P_mX'P_k-I \rightarrow I-X-P_n-I + I-P_mX'P_k^{\bullet}$

$I-XP_n^{\bullet} + M \rightarrow I-XP_{n+1}^{\bullet}$

<u>reactions of $I-P_nX^{\bullet}$</u>

$I-P_nX^{\bullet} + I-X'-I \rightarrow I-XP_n-I + I-X'^{\bullet}$

$I-P_nX^{\bullet} + D-X'-I \rightarrow I-XP_n-I + D-X'^{\bullet}$

$I-P_nX^{\bullet} + I-X'P_m-I \rightarrow I-XP_n-I + I-X'P_m^{\bullet}$

$I-P_nX^{\bullet} + I-X'P_m-I \rightarrow I-XP_n-I + I-P_mX'^{\bullet}$

$I-P_nX^{\bullet} + D-X'P_m-I \rightarrow I-XP_n-I + D-X'P_m^{\bullet}$

$I-P_nX^{\bullet} + I-X'P_m-D \rightarrow I-XP_n-I + D-P_mX'^{\bullet}$

$I-P_nX^{\bullet} + D-P_mX'P_k-I \rightarrow I-XP_n-I + D-P_mX'P_k^{\bullet}$

$I-P_nX^{\bullet} + I-P_mX'P_k-I \rightarrow I-XP_n-I + I-P_mX'P_k^{\bullet}$

$I-P_nX^{\bullet} + M \rightarrow I-P_nXP_1^{\bullet}$

<u>reactions of $D-P_nX^{\bullet}$</u>

$D-XP_n^{\bullet} + I-X'-I \rightarrow D-XP_n-I + I-X'^{\bullet}$

$D-XP_n^{\bullet} + D-X'-I \rightarrow D-XP_n-I + D-X'^{\bullet}$

$D-XP_n^{\bullet} + I-X'P_m-I \rightarrow D-XP_n-I + I-X'P_m^{\bullet}$

$D-XP_n^{\bullet} + I-X'P_m-I \rightarrow D-XP_n-I + I-P_mX'^{\bullet}$

$D-XP_n^{\bullet} + D-X'P_m-I \rightarrow D-XP_n-I + D-X'P_m^{\bullet}$

$D-XP_n^{\bullet} + I-X'P_m-D \rightarrow D-XP_n-I + D-P_mX'^{\bullet}$

D-X$P_n^\bullet$	+	D-P_mX‘P_k-I	→	D-XP_n-I	+	D-P_mX’$P_k^\bullet$
D-X$P_n^\bullet$	+	I-P_mX‘P_k-I	→	D-XP_n-I	+	I-P_mX’$P_k^\bullet$
D-X$P_n^\bullet$	+	M	→	D-X$P_{n+1}^\bullet$		

<u>reactions of D-P_nX$P_m^\bullet$</u>

D-P_nX$P_m^\bullet$	+	I-X‘-I	→	D-P_nXP_m-I	+	I-X’$^\bullet$
D-P_nX$P_m^\bullet$	+	D-X‘-I	→	D-P_nXP_m-I	+	D-X’$^\bullet$
D-P_nX$P_m^\bullet$	+	I-X‘P_k-I	→	D-P_nXP_m-I	+	I-X’$P_k^\bullet$
D-P_nX$P_m^\bullet$	+	I-X‘P_k-I	→	D-P_nXP_m-I	+	I-P_kX’$^\bullet$
D-P_nX$P_m^\bullet$	+	D-X‘P_k-I	→	D-P_nXP_m-I	+	D-X’$P_k^\bullet$
D-P_nX$P_m^\bullet$	+	I-X‘P_k-D	→	D-P_nXP_m-I	+	D-P_kX’$^\bullet$
D-P_nX$P_m^\bullet$	+	D-P_lX‘P_k-I	→	D-P_nXP_m-I	+	D-P_lX’$P_k^\bullet$
D-P_nX$P_m^\bullet$	+	I-P_lX‘P_k-I	→	D-P_nXP_m-I	+	I-P_lX’$P_k^\bullet$
D-P_nX$P_m^\bullet$	+	M	→	D-P_nX$P_{m+1}^\bullet$		

<u>reactions of I-P_nX$P_m^\bullet$</u>

I-P_nX$P_m^\bullet$	+	I-X‘-I	→	I-P_nXP_m-I	+	I-X’$^\bullet$
I-P_nX$P_m^\bullet$	+	D-X‘-I	→	I-P_nXP_m-I	+	D-X’$^\bullet$
I-P_nX$P_m^\bullet$	+	I-X‘P_k-I	→	I-P_nXP_m-I	+	I-X-$P_k^\bullet$
I-P_nX$P_m^\bullet$	+	I-X‘P_k-I	→	I-P_nXP_m-I	+	I-P_kX’$^\bullet$
I-P_nX$P_m^\bullet$	+	D-X‘P_k-I	→	I-P_nXP_m-I	+	D-X’$P_k^\bullet$
I-P_nX$P_m^\bullet$	+	I-X‘P_k-D	→	I-P_nXP_m-I	+	D-P_kX’$^\bullet$
I-P_nX$P_m^\bullet$	+	D-P_lX‘P_k-I	→	I-P_nXP_m-I	+	D-P_lX’$P_k^\bullet$
I-P_nX$P_m^\bullet$	+	I-P_lX‘P_k-I	→	I-P_nXP_m-I	+	I-P_lX’$P_k^\bullet$
I-P_nX$P_m^\bullet$	+	M	→	I-P_nX$P_{m+1}^\bullet$		

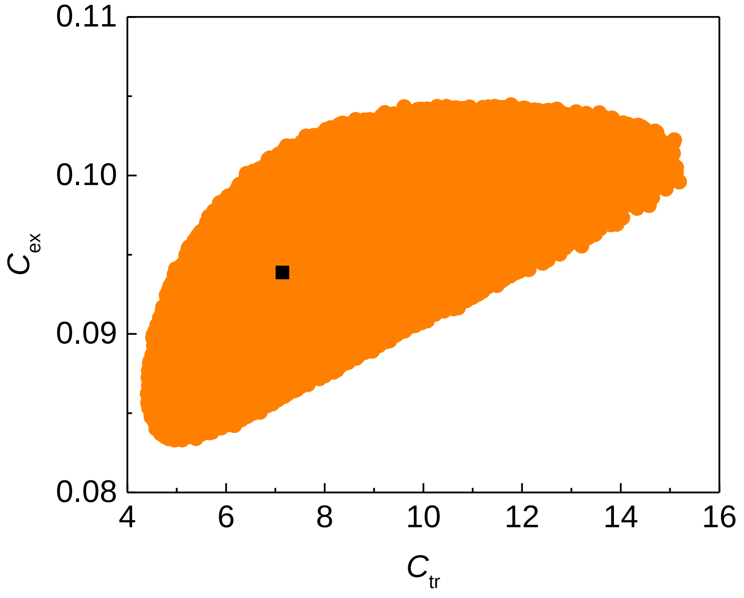

Figure S1: Confidence interval for the parameters C_{tr} and C_{ex} and optimum parameters (black).

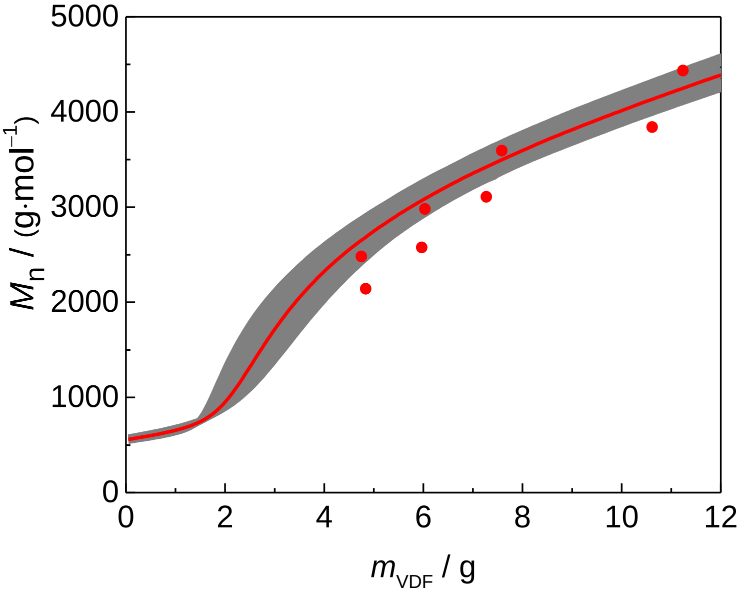

Figure S2: Sensitivity of the parameters C_{tr} and C_{ex}. The red line shows the simulation results with the optimum values and the red points the experimental data points (20 bar, 7.5 mmol I-C_4F_8-I (sample 5)). The grey band represents simulations carried out with 16 points taken from the outer sphere of the confidence interval, indicating that in all cases a good description of the experimental results is obtained.
